# SdrF, a *Staphylococcus epidermidis* Surface Protein, Contributes to the Initiation of Ventricular Assist Device Driveline–Related Infections

**DOI:** 10.1371/journal.ppat.1000411

**Published:** 2009-05-01

**Authors:** Carlos Arrecubieta, Faustino A. Toba, Manuel von Bayern, Hirokazu Akashi, Mario C. Deng, Yoshifumi Naka, Franklin D. Lowy

**Affiliations:** 1 Division of Infectious Diseases, Department of Medicine, College of Physicians and Surgeons, Columbia University, New York, New York, United States of America; 2 Division of Cardiology, Department of Medicine, College of Physicians and Surgeons, Columbia University, New York, New York, United States of America; 3 Department of Surgery, New York Presbyterian Hospital, Columbia University, New York, New York, United States of America; 4 Department of Pathology, College of Physicians and Surgeons, Columbia University, New York, New York, United States of America; Dartmouth Medical School, United States of America

## Abstract

*Staphylococcus epidermidis* remains the predominant pathogen in prosthetic-device infections. Ventricular assist devices, a recently developed form of therapy for end-stage congestive heart failure, have had considerable success. However, infections, most often caused by *Staphylococcus epidermidis*, have limited their long-term use. The transcutaneous driveline entry site acts as a potential portal of entry for bacteria, allowing development of either localized or systemic infections. A novel *in vitro* binding assay using explanted drivelines obtained from patients undergoing transplantation and a heterologous lactococcal system of surface protein expression were used to identify *S. epidermidis* surface components involved in the pathogenesis of driveline infections. Of the four components tested, SdrF, SdrG, PIA, and GehD, SdrF was identified as the primary ligand. SdrF adherence was mediated via its B domain attaching to host collagen deposited on the surface of the driveline. Antibodies directed against SdrF reduced adherence of *S. epidermidis* to the drivelines. SdrF was also found to adhere with high affinity to Dacron, the hydrophobic polymeric outer surface of drivelines. Solid phase binding assays showed that SdrF was also able to adhere to other hydrophobic artificial materials such as polystyrene. A murine model of infection was developed and used to test the role of SdrF during *in vivo* driveline infection. SdrF alone was able to mediate bacterial adherence to implanted drivelines. Anti-SdrF antibodies reduced *S. epidermidis* colonization of implanted drivelines. SdrF appears to play a key role in the initiation of ventricular assist device driveline infections caused by *S. epidermidis*. This pluripotential adherence capacity provides a potential pathway to infection with SdrF-positive commensal staphylococci first adhering to the external Dacron-coated driveline at the transcutaneous entry site, then spreading along the collagen-coated internal portion of the driveline to establish a localized infection. This capacity may also have relevance for other prosthetic device–related infections.

## Author Summary

This study investigates the manner in which heart pumps become infected. Infections involving the driveline, the cord that connects the pump to its external power source, are a major complication of implantation of these devices. In this study, we examined why *Staphylococcus epidermidis*, a bacteria that is commonly found on the skin, is responsible for the majority of these infections. The ability of different surface molecules of *S. epidermidis* to attach to drivelines removed from patients undergoing transplantation was tested. SdrF, one of the *S. epidermidis* surface proteins studied, showed the highest adherence to the drivelines. This binding appeared to involve attachment of the B subunit of SdrF to collagen, a molecule that coats the driveline following implantation. SdrF also mediated attachment to the non-implanted Dacron material that is used to coat the drivelines. A mouse driveline infection model also demonstrated that SdrF was sufficient to initiate a driveline infection. Driveline infection can therefore be hypothesized to occur via: 1) skin colonization by *S. epidermidis*; 2) adherence of SdrF-positive *S. epidermidis* to the Dacron surface at the skin entry site; 3) migration of bacteria along the driveline; 4) adherence to the internal driveline via the collagen coating its surface; and 5) initiation of infection.

## Introduction

*Staphylococcus epidermidis* is a major cause of prosthetic device infections. This capacity appears due to its ability to colonize both the skin as commensal flora and prosthetic materials via its ability to adhere to device surfaces and form biofilms [1]–[3]. Ventricular assist devices (VADs) are relatively new cardiovascular prostheses that have become a major form of therapy for patients with end-stage congestive heart failure. Originally developed as a bridge to cardiac transplantation, they are increasingly used as “destination therapy” (*e.g.*, a means to improve survival and quality of life in patients with heart failure refractory to medical therapy) [4]. However, an important limitation to the use of VADs has been the high incidence of device-related infections, which occur in 18%–59% of patients [5]–[8]. These infections can affect different components of the device, such as the surgical site, the device pocket or the device itself and pose a major threat to survival since eradication usually requires device removal [5], [9]–[11].

Although the clinical features of VAD-associated infections have been well described, less attention has been devoted to their pathogenetic processes [5],[7],[12]. The driveline (DL) is the most common site of VAD-related infections [6],[9],[13],[14]. This is likely due to the transcutaneous entry site, continued exposure to commensal flora and the prolonged VAD implantation times [15]. DL infections may remain localized to the entry site or progress, via an ascending infection, to cause further complications including sepsis and endocarditis [7],[8],[16]. DLs are coated with a layer of highly textured polyethylene terephthalate (Dacron), a polyester material that facilitates integration into the skin and soft tissues, protecting against bacterial entry. However, mobility of the DL frequently impedes healing and increases the possibility of colonization, biofilm formation and infection [11].

DL infections, like other prosthetic device infections, are most often caused by *S. epidermidis* [11]. The critical step in the initiation of these infections is bacterial colonization of host structures. Staphylococci, including both *Staphylococcus aureus* and *S. epidermidis*, possess a wide range of structurally-related surface proteins with redundant adhesive properties, many of them belonging to a structurally related family of microbial surface components recognizing adhesive matrix molecules (MSCRAMMs), that facilitate this initial colonization step [17]–[21].

The goal of this study was to investigate the pathogenesis of this unique prosthetic device infection as well as to establish a biological model for the study of other prosthetic device infections. By examining the adherence of *S. epidermidis* proteins to the surface of explanted VAD drivelines and to implanted DLs in a murine model of infection we found that SdrF (GenBank #AF245041), a protein without a previously identified role in foreign body infections, mediates *S. epidermidis* adherence to both the external and internal host-tissue coated components of the DL.

## Results

### Histological analysis of explanted DLs

**Figure 1:**
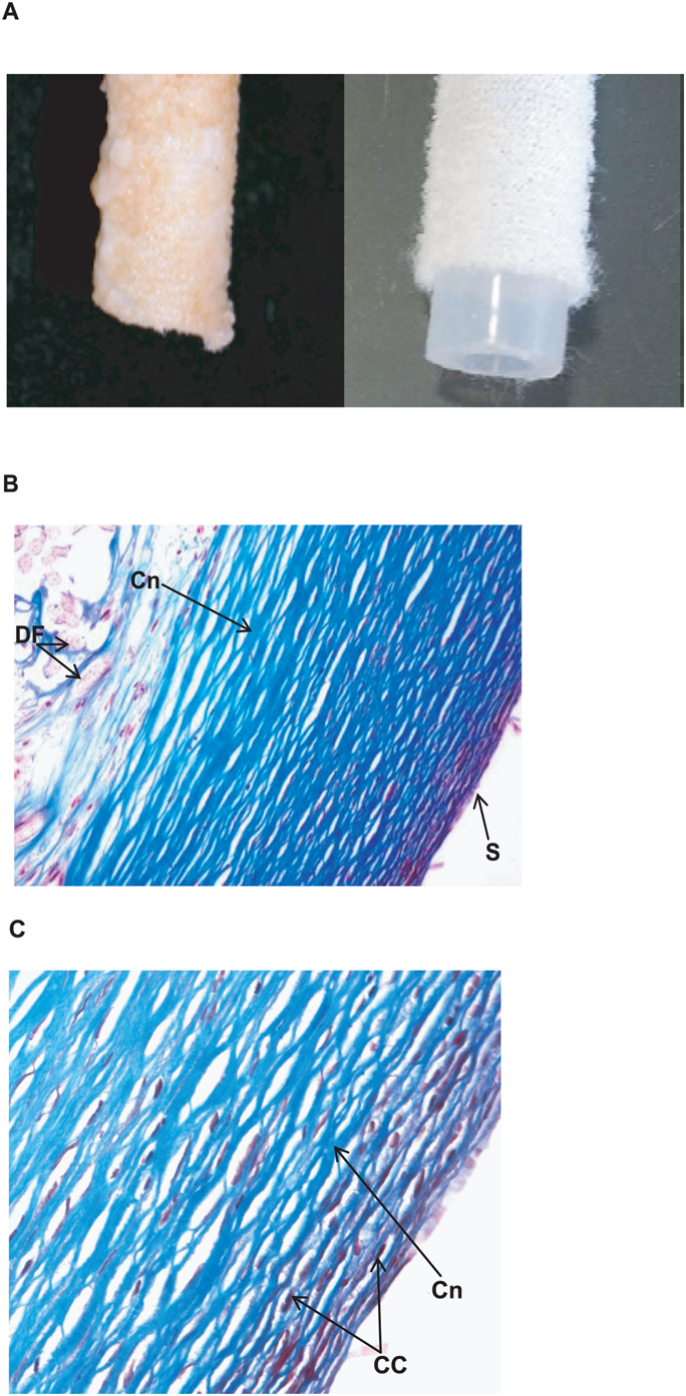
Histological analysis of host components coating an explanted driveline (DL). (A) Comparison of the external appearance of a typical explanted DL (left panel) and a non-implanted DL (right panel). (B) Low-resolution view (100×) of a Trichrome-stained section (6 µm) of a 20 wk–implanted DL showing the thick, fibrous collagen layer deposited on the Dacron fibers. (C) Detailed view (400×) of host cellular components interspersed among collagen fibers. Collagen stains blue with Trichrome stain. DF, Dacron fibers; Cn, collagen fibers; S, coated DL surface; CC, cellular components.

Five DLs from Heartmate II VADs, implanted for different periods of time (13–30 weeks), were obtained from patients undergoing removal of the device for transplantation. During implantation, host matrix components are deposited on the surface of the DL modifying its appearance with respect to non-implanted DLs (Figure 1A) and mostly resulting in the DL material, Dacron, no longer being accessible to the bacteria. Trichrome staining of the outer surface of the implanted DLs demonstrated that collagen was the major host component on the surface of the internal DL (Figure 1B). Fibroblasts were also detected interspersed within the collagen fibers (Figure 1C).

### Adherence of *Lactococcus lactis* recombinant strains expressing *S. epidermidis* surface components to explanted DLs

**Figure 2:**
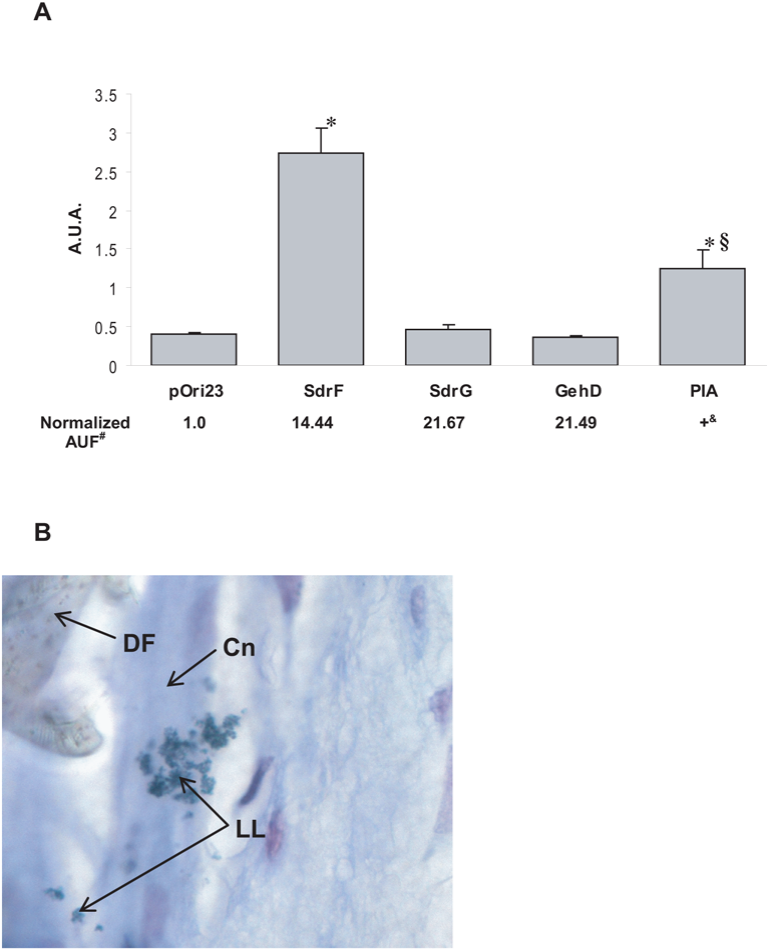
Adherence of *S. epidermidis* surface molecules to explanted DLs. (A) Cell wall–associated SdrF mediates *L. lactis* cells adherence to explanted DLs. PIA production by *L. lactis* elicits lower levels of adherence than SdrF. A.U.A., arbitrary units of adherence; *, *P*<0.05 when compared to pOri23 (control); §, *P*<0.05 when compared to SdrF. Recombinant phenotype analyzed by flow cytometry is shown below. A.U.F., arbitrary units of fluorescence calculated by multiplying the percentage of positive (fluorescent) events by the average fluorescence per event. #, normalized A.U.F. was calculated assigning an arbitrary value of 1.0 to the control strain (pOri23) incubated with the appropriate polyclonal antibody for each recombinant protein. &, PIA phenotype was determined by biofilm production assessment as previously described [23]. (B) Gram stain view (1000×) of *L. lactis* (pOri-SdrF) cells adherent to an explanted DL. DF, Dacron fibers; Cn, collagen fibers; LL, *L. lactis* cells.

The genes coding for likely *S. epidermidis* ligands were selected for comparison [2]. SdrF was cloned in *L. lactis* MG1363. Two additional *S. epidermidis* surface proteins, SdrG (GenBank #AF245042) and GehD (GenBank #AF090142), as well as the *icaADBC* operon (GenBank #DQ149646), responsible for the synthesis of polysaccharide intercellular adhesin (PIA) and subsequent biofilm formation, were cloned in *L. lactis* NZ9000 (Table 1). Assessment of production and estimation of relative amounts of surface-bound recombinant SdrF, GehD and SdrG in these *L. lactis* strains were demonstrated by flow cytometry. All three recombinant proteins were exported and anchored to the lactococcal cell wall (Figure 2A). Production of PIA was assessed in a microplate biofilm assay as previously described (data not shown) [22]. Subsequently, the binding capacity of these *L. lactis* strains, as well as that of *L. lactis* pOri-SdrF, which produces the *S. epidermidis* collagen-binding protein SdrF [23], to explanted DLs was measured.

**Table 1:** Recombinant plasmids used in this study.

Plasmid Name	Cloned DNA	Oligonucleotides^a^ (Endonuclease)	*S. epidermidis* Source Strain	Phenotype
pOri-sdrG	*sdrG*	aaaggatcccattgaaatagtcaaagaaaaggag (*Bam*HI)	K28	SdrG+ [38]
		ttcctgcagcatatctgcttctttctacaaatttc (*Pst*I)		
pOri-gehD	*gehD*	aaaggatccggggaaaaaacttatagaggtgat (*Bam*HI)	9	GehD+ [20]
		aaaggatccgtaatcatatatttaatgaggatg (*Bam*HI)		
pOri-icaO-47	*icaADBC*	cctctgcagcgaaaggtaggtgaaaaaatgc (*Pst*I)	O-47	PIA+ [39] (Biofilm)
		catctgcaggccatagcttgaataagggac (*Pst*I)		
pOri-SdrF	*sdrF*	aaaggatccctggaggtatagtatgaaaaagag (*Bam*HI)	9491	SdrF+ [23]
		aaactgcagctatttttctttattatcttttttacgacgtcttcc (*Pst*I)		
pOri-SdrFA18	*sdrF* minus A domain	aaaggatccctggaggtatagtatgaaaaagag (*Bam*HI)	9491	SdrF lacking A domain [23]
		attccatggtgagttttcattatcacgactacc (*Nco*I)		
		For construction details see [23]		
pOri-SdrFN856	*sdrF* minus B domain	atagataatggttattttgacccatggtcagacagtg (*Nco*I)	9491	SdrF lacking B domain [23]
		aaactgcagctatttttctttattatcttttttacgacgtcttcc (*Pst*I)		
		For construction details, see [23].		

a Restriction sites created are underlined.

The *L. lactis* strain producing SdrF on its surface adhered significantly better to explanted DLs than the control strain harboring the vector pOri23 alone (Figure 2A). Similarly, the biofilm-producing *L. lactis* pOri-icaO-47 strain showed a significant increase in DL adherence, although this adherence was significantly lower than that of SdrF (Figure 2A). The adherence of SdrG, a fibrinogen-binding surface protein, was no different than the control. Surprisingly, GehD, a collagen-binding protein, did not demonstrate significant adherence to the collagen-coated DLs when compared with the control (Figure 2A). Gram stain of explanted DL sections incubated with *L. lactis* harboring pOri-SdrF showed bacteria attached to the collagen fibers coating the Dacron-textured surface (Figure 2B).

### Involvement of the B domain of SdrF in adherence to explanted DLs

**Figure 3:**
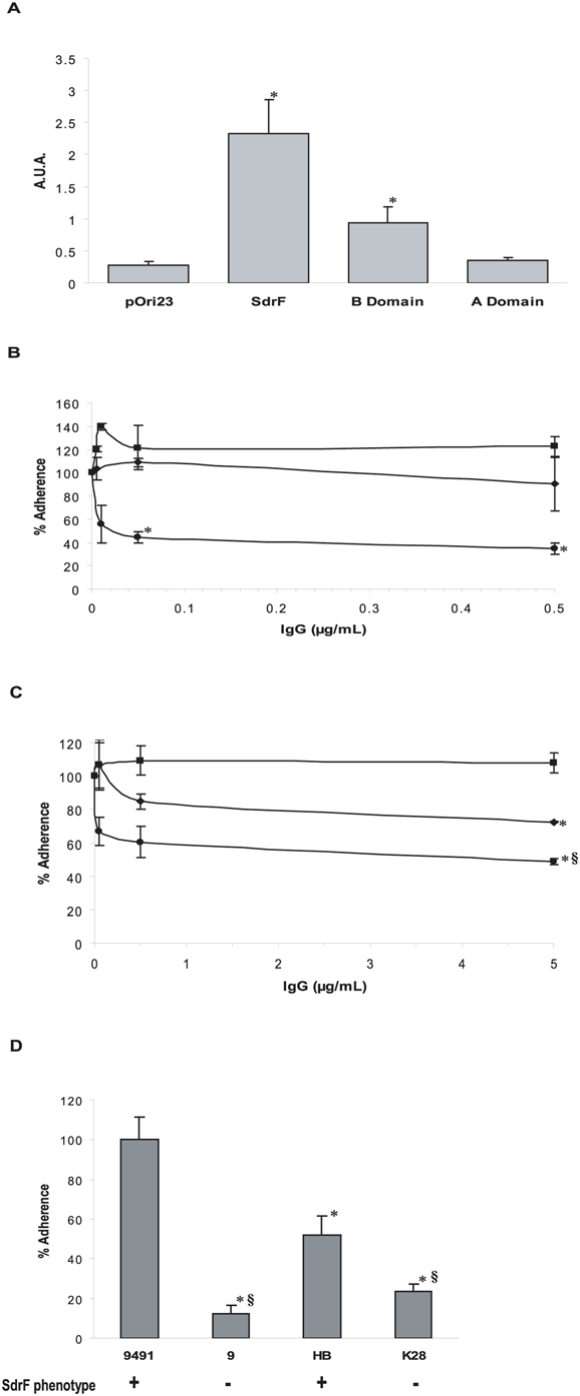
Adherence of SdrF domains and *S. epidermidis* to explanted DLs. (A) Binding of *L. lactis* strains producing truncated forms of SdrF to explanted DLs. A.U.A., arbitrary units of adherence; *, *P*<0.05 when compared to pOri23 (control). pOri23, *L. lactis* control strain; SdrF, *L. lactis* expressing surface-exposed full-length SdrF; B-domain, *L. lactis* expressing surface-exposed truncated SdrF containing the B domain; A-domain, *L. lactis* expressing surface-exposed truncated SdrF containing the A domain. (B) Increasing concentrations of antibodies were pre-incubated with *L. lactis* (pOri-SdrF) cells before incubation with explanted DL disks. ▪, pre-immune IgGs; ♦, anti-rASdrF IgGs; •, anti-rBSdrF IgGs; *, *P*<0.05 when compared to the control experiment (pre-immune IgGs). (C) Increasing concentrations of antibodies were pre-incubated with *S. epidermidis* 9491 cells prior to adding explanted DL disks. ▪, pre-immune IgGs; ♦, anti-rASdrF IgGs; • anti-rBSdrF IgGs; *, *P*<0.05 when compared to the control experiment (pre-immune IgGs). §, *P*<0.05 when compared to anti-rASdrF IgGs. (D) Comparison of adherence of four *S. epidermidis* strains to explanted DL disks. *S. epidermidis* 9491 was assigned a 100% binding capacity. SdrF phenotype is shown below. *, *P*<0.05 when compared to 9491. §, *P*<0.05 when compared to HB.

SdrF contains four regions: a putative ligand-binding A domain (rASdrF), the recently identified collagen-binding B domain (rBSdrF), a serine-aspartate repeat region, which is thought to act as a stem spanning the staphylococcal cell wall therefore allowing domains A and B to protrude from the cell surface, and a C-terminal region which anchors the protein to the peptidoglycan. Two *L. lactis* recombinant strains that produce truncated cell wall-associated forms of SdrF lacking either the A or B domains were constructed in an earlier study (*L. lactis* pOri-SdrFNA18 and pOri-SdrFN856 respectively), where SdrF was found to bind collagen type I via its B domain [23]. Binding of *L. lactis* strains producing either A or B domains of SdrF on their surface to explanted DLs was compared to that of the full length SdrF. *L. lactis* cells producing the surface-associated B domain adhered significantly better to explanted DLs than to either the control strain or to cells producing the A domain on their surface (Figure 3A).

Purified antibodies directed against either the A or the B domain of SdrF were used to further assess the involvement of both regions in DL binding [23]. Control IgGs as well as anti-rASdrF IgGs did not affect *L. lactis* SdrF-mediated binding to explanted DLs. In contrast, the presence of anti-rBSdrF IgGs at low concentrations (50 ng/mL) caused a significant reduction in binding (Figure 3B).

### Adherence of *S. epidermidis* to explanted DLs

*S. epidermidis*, like *S. aureus*, has redundancy in its adherence capacity with more than one protein often mediating adherence to the same host molecule [20], [23]–[26]. We therefore investigated the effect of antibodies directed against different regions of SdrF on adherence of *S. epidermidis* to explanted DLs. Anti-rBSdrF caused a significant reduction in the SdrF-prototype *S. epidermidis* 9491 [18] binding to explanted DLs (Figure 3C). In addition, *S. epidermidis* 9491 cells pre-incubated with anti-rASdrF also showed reduced adherence, although this reduction was significantly less pronounced than in cells pre-incubated with anti-rBSdrF (Figure 3C). Pre-immune IgGs had no effect on *S. epidermidis* adherence. Furthermore, SdrF-positive strains *S. epidermidis* 9491 and HB adhered with higher affinity to the explanted DLs than the SdrF-negative strains 9 and K28 (Figure 3D).

### Adherence of SdrF to textured Dacron surfaces

**Figure 4:**
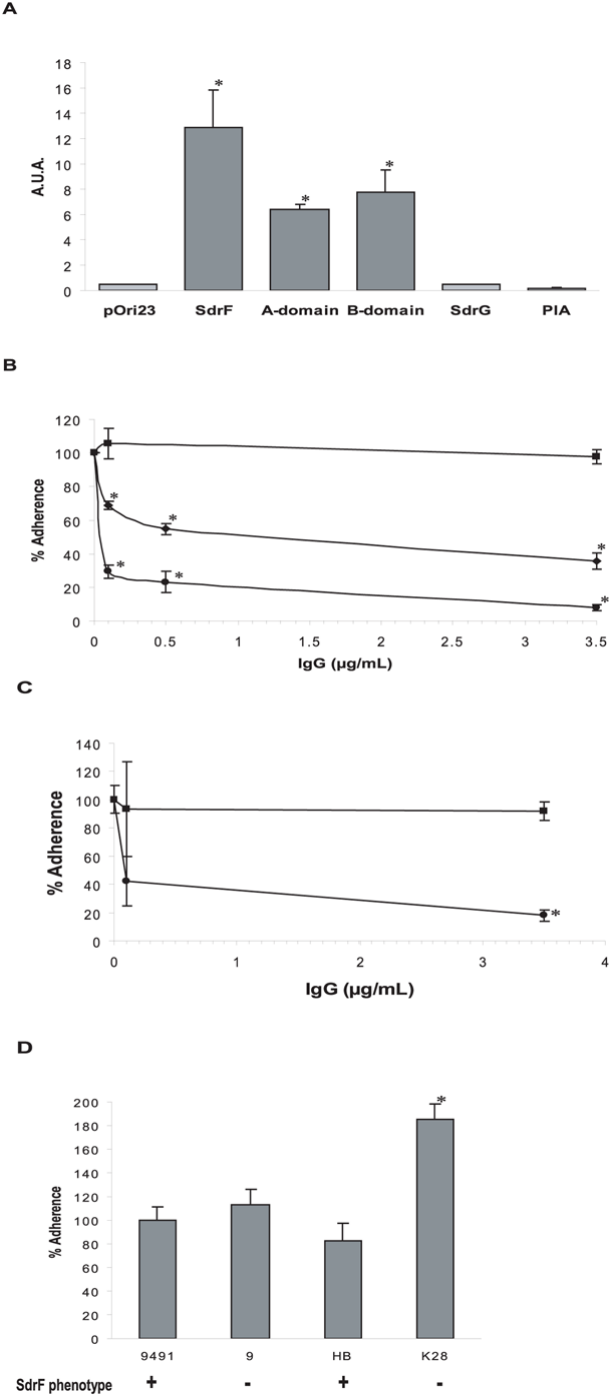
Binding of *S. epidermidis* factors to Dacron DLs. (A) Adherence of *L. lactis* strains producing recombinant cell wall–associated factors to Dacron-textured DLs. A.U.A., arbitrary units of adherence; *, *P*<0.05 when compared to pOri23 (control). (B) Increasing concentrations of antibodies were pre-incubated with *L. lactis* (pOri-SdrF) before addition of non-implanted DL pieces. ▪, pre-immune IgGs; ♦, anti-rASdrF IgGs; •, anti-rBSdrF IgGs; *, *P*<0.05 when compared to the control experiment (no antibody). (C) Increasing concentrations of antibodies were pre-incubated with *S. epidermidis* 9491 cells prior to adding Dacron-textured DL disks. ▪, pre-immune IgGs; •, mixture (1∶1) of anti-rASdrF and anti-rBSdrF IgGs; *, *P*<0.05 when compared to the control experiment (pre-immune IgGs). (D) Comparison of adherence of four *S. epidermidis* strains to Dacron-textured DL disks. *S. epidermidis* 9491 was assigned a 100% binding capacity. SdrF phenotype is shown below. *, *P*<0.05 when compared to 9491.

The highly textured Dacron surface covering the DL promotes tissue integration as a way to diminish the occurrence of infections. However, the effect of this material on bacterial adhesion has not been investigated. The binding capacity of *S. epidermidis* proteins to non-implanted DLs was measured using the different *L. lactis* constructs. *L. lactis* cells containing SdrF on their surface adhered at a significantly higher rate than the control strain (Figure 4A). Similarly, cells producing truncated forms of SdrF lacking either the A or the B domains were able to adhere to Dacron-textured surfaces (Figure 4A). The presence of either surface-associated SdrG or PIA did not mediate adherence to non-implanted DLs (Figure 4A).

Anti-rASdrF and anti-rBSdrF IgGs separately caused a significant reduction in *L. lactis* (pOri-SdrF) adherence to non-implanted DLs whereas control pre-immune IgGs did not affect binding (Figure 4B). The role of SdrF in *S. epidermidis* adherence to Dacron was further analyzed by assessing the effect of anti-rASdrF and anti-rBSdrF IgGs on *S. epidermidis* binding to non-implanted DLs. A solution (1∶1) of these two anti-SdrF antibodies significantly reduced adherence of *S. epidermidis* 9491 cells to Dacron whereas control pre-immune IgGs did not cause any apparent effect (Figure 4C). In addition we compared the relative adherence of four *S. epidermidis* strains. While all *S. epidermidis* strains were found to adhere to Dacron, K28, an SdrF-negative strain, showed a significantly higher adherence level (Figure 4D).

### SdrF-mediated adherence to polystyrene surfaces

**Figure 5:**
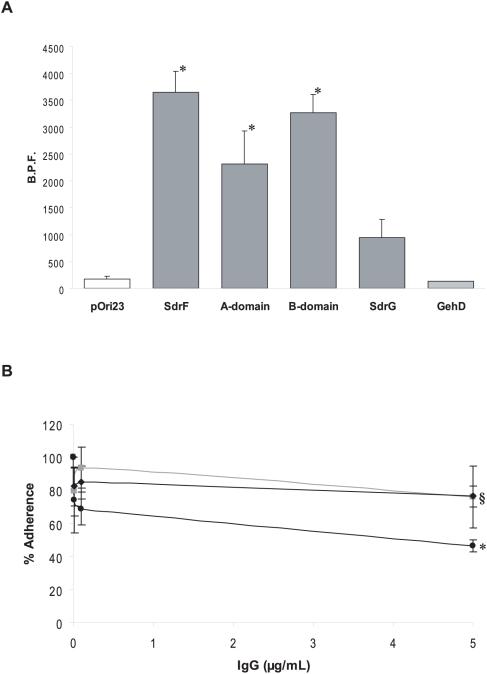
Adherence of SdrF to polystyrene surfaces. (A) Binding of *L. lactis* strains containing *S. epidermidis* recombinant surface proteins was tested on polystyrene microplate wells. B.P.F., bacteria per field (400×magnification); *, *P*<0.05 when compared to pOri23 (control). (B) Increasing concentrations of antibodies were pre-incubated with *L. lactis* (pOri-SdrF) before exposure to a polystyrene surface. ▪, pre-immune IgGs; ♦, anti-rASdrF IgGs; •, anti-rBSdrF IgGs; *, *P*<0.05 when compared to the control experiment (no antibody). §, *P*<0.05 (for Anti-rASdrF) when compared to the control experiment (no antibody).

In order to further assess whether the textured nature of the material was a factor in adherence to the Dacron, SdrF binding to a flat polymer surface was measured. SdrF was found to mediate *L. lactis* adherence to polystyrene surfaces whereas SdrG and GehD had limited binding (Figure 5A). In addition the effect of anti-SdrF antibodies on binding to polystyrene was assessed. Both anti-rASdrF and anti-rBSdrF IgGs effectively reduced SdrF attachment (Figure 5B). Pre-immune control IgGs again had no effect on adhesion to polystyrene (Figure 5B).

### SdrF mediates *in vivo* adherence to transcutaneous DLs

**Figure 6:**
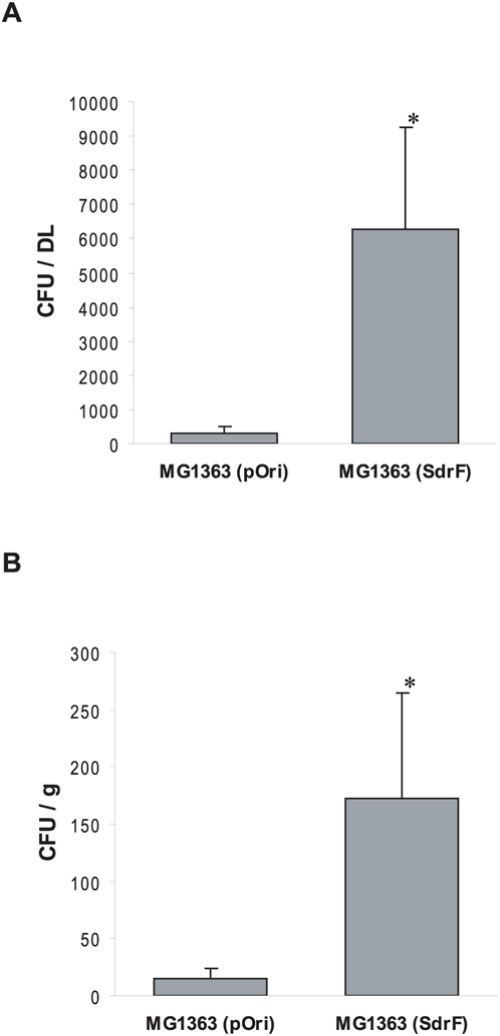
*In vivo* murine model of DL infection measuring the ability of *L. lactis* expressing SdrF to establish a DL infection. (A) Adherence of control (MG1363 pOri) or SdrF-expressing *L. lactis* (MG1363 SdrF) to DL following implantation and infection. *, *P*<0.05. (B) Adherence of control (MG1363 pOri) or SdrF-expressing *L. lactis* (MG1363 SrdF) to tissue surrounding the DL following implantation and infection. *, *P*<0.05.

**Figure 7:**
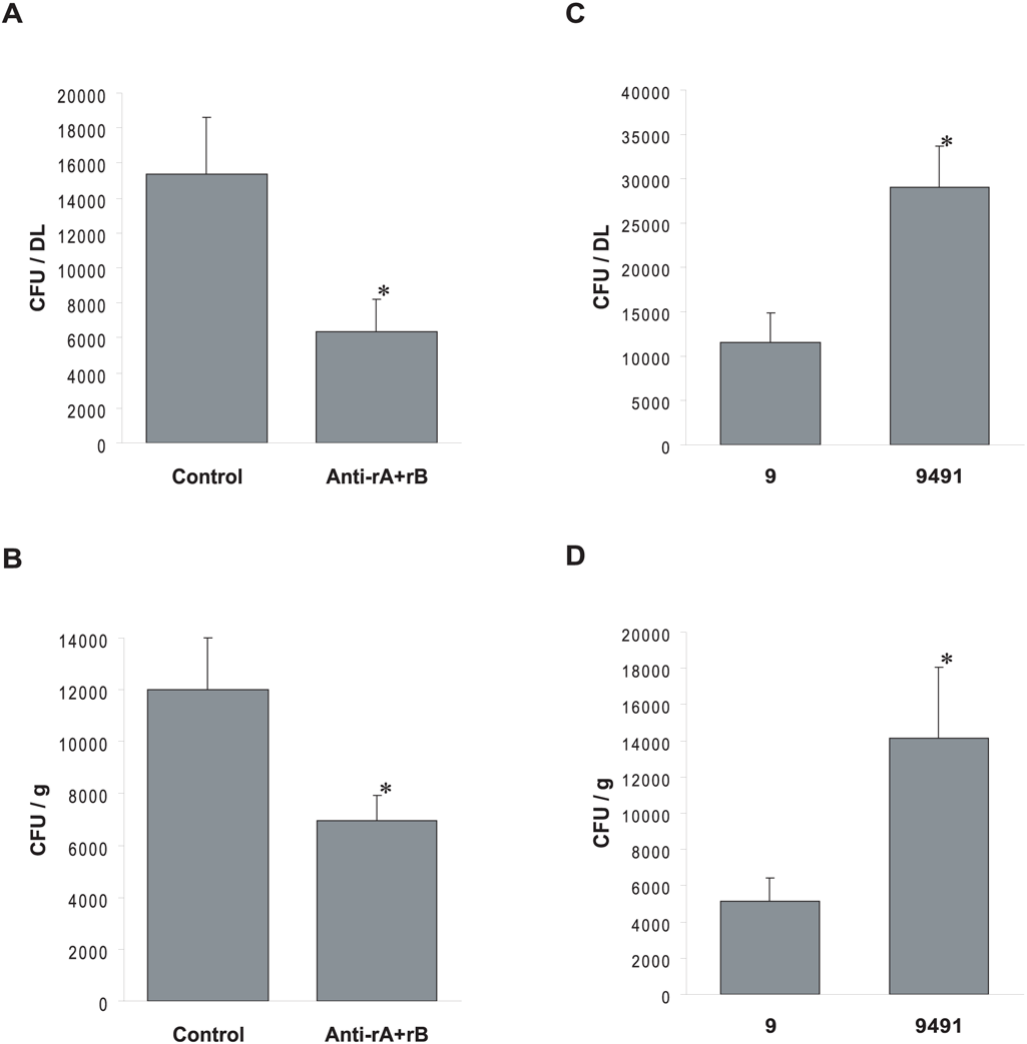
*In vivo* murine model of DL infection measuring the ability of *S. epidermidis* to establish a DL infection. (A,B) 3.75 µg of either pre-immune (Control) IgGs or a mixture (1∶1) of anti-rASdrF and anti-rBSdrF IgGs (Anti-rA+rB) were pre-incubated with *S. epidermidis* 9491 cells before infection. (A) Effect of anti-SdrF IgGs on the *in vivo* adherence of *S. epidermidis* 9491 to DL. (B) Effect of anti-SdrF IgGs on the *in vivo* adherence of *S. epidermidis* 9491 to tissue surrounding the DL following implantation and infection. (C,D) Comparison of *in vivo* colonization between *S. epidermidis* strains 9491 (SdrF +) and 9 (SdrF −) to DL (C) and surrounding tissue (D). *, *P*<0.05.

A recently developed transcutaneous DL murine model was used to further assess the role of SdrF in DL infections [27]. Skin surrounding the DL entry point was inoculated with *L. lactis* pOri23 (control; n = 15) or *L. lactis* pOri-SdrF cells (n = 15). Both DLs and a sample of surrounding tissue were removed 48 hours after infection. The presence of SdrF on the lactococcal cell surface caused a significant increase in the number of bacterial cells attached to the internal portion of the DL as well as to the surrounding tissue with respect to the controls (Figure 6A and 6B). In order to assess the role of wild type SdrF in DL colonization by *S. epidermidis*, a mixture (1∶1) of antibodies directed against the A and B domains were pre-incubated with *S. epidermidis* 9491 cells prior to DL inoculation (n = 15). Significantly fewer *S. epidermidis* cells pre-incubated with anti-SdrF IgGs than cells pre-incubated with control IgGs colonized the DL and the surrounding tissue (Figure 7A and 7B). Moreover, SdrF-positive *S. epidermidis* 9491 was found to adhere significantly better to both DLs and tissue than the previously described SdrF-negative *S. epidermidis* 9 (Figure 7C and 7D).

## Discussion

*S. epidermidis* prosthetic device infections are in general initiated by surface proteins that mediate bacterial attachment directly to the synthetic surface or indirectly via host components, such as fibrinogen, that are deposited on the surface of foreign material [25],[26]. This is the first study to examine the role of *S. epidermidis* surface components in the pathogenesis of DL infections. After implantation, the highly textured Dacron driveline surface is coated by host matrix constituents, most notably collagen and cellular components, including fibroblasts and/or myofibroblasts.

To overcome the inherent redundancy found in *S. epidermidis* adhesins we used a heterologous lactococcal expression system combined with a screening assay to test for the adherence of *S. epidermidis* surface proteins to DL surfaces. The intrinsic heterogeneity of the implanted DL surface, as well as the variation due to implantation time resulted in the qualitative nature of our assay. We found that SdrF mediated adherence to explanted DL disks, via its attachment to collagen fibers present on the DL surface. The formation by *L. lactis* of PIA-dependent biofilm also increased the number of bacteria bound to DL disks. This binding was significantly lower than that of SdrF, and a nonspecific interaction due to the clumping effect of the biofilm cannot be ruled out.

SdrF consists of four main components: a ligand binding domain A, a collagen binding domain B, the serine-aspartate repeat region and the C-terminal region which anchors the protein to the peptidoglycan [18],[19],[23]. Using previously described recombinant *L. lactis* strains [23] we demonstrated that the B domain was responsible for SdrF binding to explanted DLs. This interaction seemed to be specific since antibodies against the B domain reduced DL binding in a dose-dependent, saturable manner.

Binding studies using *S. epidermidis*, where other adhesins might also contribute to adherence, further confirmed the role of SdrF. Even at relatively high antibody concentrations, the reduction in *S. epidermidis* binding did not exceed 50%, further reinforcing the notion that other adhesins are likely to be involved in DL binding. One candidate would be the surface protein GehD, which was previously shown to have collagen-binding activity [20]. However, this protein did not cause any adherence when present on the *L. lactis* or the *S. epidermidis* cell wall. Comparison of adherence between *S. epidermidis* strains HB and K28, two strains differing in their SdrF phenotype, also suggested that SdrF plays an important role in *S. epidermidis* adherence to DLs. However, binding differences due to differential production of other surface proteins cannot be ruled out.

The highly textured Dacron outer layer of VAD drivelines promotes tissue integration and wound healing [15]. Rapid attachment of the bacteria to implanted surfaces appears to be the first step towards biofilm formation and subsequent colonization by *S. epidermidis* [3]. The *S. epidermidis* autolysin AtlE has been shown to mediate initial attachment to polystyrene surfaces via hydrophobic interactions, although it is unclear whether the autolysin plays a direct role in this event [25].

We investigated the interaction between SdrF and the polyester surface of the DL. SdrF bound with high affinity to exposed Dacron layer of the non-implanted DL. This phenomenon persisted when either the A or B domains were absent, suggesting that this interaction might be due to hydrophobic forces rather than to conformational features of the polypeptide. Adherence did not occur when other *S. epidermidis* surface factors such as SdrG and PIA were produced by *L. lactis* cells, suggesting that this is SdrF-specific. Interestingly, adherence to the non-implanted DLs was significantly greater than binding to collagen-coated explanted DLs. Anti-SdrF antibodies reduced adherence to Dacron, supporting the notion that SdrF plays a role in the initial attachment to the highly textured surface of DLs. SdrF-defective *S. epidermidis* strains 9 and K28 showed high adherence levels to Dacron, with K28 level being even higher than 9491. These observations suggest that other surface components also play a role in initial rapid attachment to implanted DLs.

We further investigated the capacity of SdrF to adhere to hydrophobic polymeric surfaces by testing the ability of *L. lactis* (pOri-SdrF) to adhere to flat polystyrene surfaces. The results demonstrated that SdrF also mediated binding to flat polystyrene surfaces. In addition, substantial amounts of antibodies against the A and B domains significantly reduced this binding.

**Figure 8:**
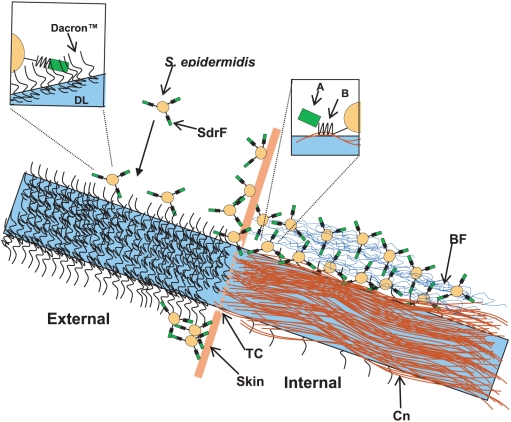
Model of attachment of *S. epidermidis* to an implanted DL. A model illustrating the potential role of SdrF in *S. epidermidis* colonization and infection of a transcutaneous driveline (DL). SdrF mediates the initial attachment of *S. epidermidis* found colonizing the skin to Dacron fibers. Bacteria cross the transcutaneous site (TC) and adhere via the SdrF B domain to collagen fibers (Cn) deposited on the internal DL surface, leading to subsequent biofilm (BF) formation, colonization, and potential infection.

Our results suggest that SdrF mediates binding to synthetic materials as well as to collagen-covered surfaces. This ability to directly mediate adhesion to synthetic materials and extracellular matrix components has been previously observed in two cell wall-associated proteins from *Erysipelothrix rhusiopathiae*, a Gram-positive animal and human pathogen [28]. SdrF appears to play a key role in the initial attachment of *S. epidermidis* to newly implanted DLs. We speculate that this process occurs in a stepwise fashion with SdrF mediating attachment of the skin commensal *S. epidermidis* to the uncoated Dacron at the DL exit site. Although unproven as yet, the bacteria may then migrate up the device by forming a biofilm and attach to the collagen-coated internal DL component (Figure 8).

To test this hypothesis we used a recently described murine VAD DL model [27]. After two days we found *L. lactis* cells producing SdrF in the internal (subcutaneous) part of the DL as well as in the surrounding muscle. We also observed that blocking cell surface-exposed SdrF with antibodies against the A and B domains reduced the ability of *S. epidermidis* to colonize both the DL and the surrounding tissue. Furthermore we found that SdrF-positive strain 9491 colonized both DL and tissue significantly better than SdrF-negative *S. epidermidis* strain 9, suggesting that SdrF might be responsible for the differential adherence. However, due to the non-isogenic nature of these two *S. epidermidis* strains, involvement of other surface factors cannot be ruled out.

The interaction of *S. epidermidis* SdrF with the DL material has a broader biological role than the VAD infections alone since other prosthetic material including tunneled intravenous catheters also utilize Dacron to foster tissue integration [29]. Future studies are needed to determine in a more precise manner the nature and characteristics of the interactions between SdrF and artificial surfaces as well as host factors. However, the high affinity for both Dacron and polystyrene demonstrated in this study strongly suggests a significant biological role in the pathogenesis of infections of indwelling medical devices by *S. epidermidis*. The strategy used in this study, *i.e.* the use of explanted devices, combined with heterologous expression systems, has proven to be a valid and useful *in vitro* approach to the study of bacterial adhesins in these complex interactions [12],[30],[31].

## Materials and Methods

### Ethics statement

All procedures were performed in accordance with protocols approved by the Institutional Animal Care and Use Committee at Columbia University. This study was reviewed and approved by the Columbia University Institutional Review Board.

### Bacterial strains, plasmids, and reagents

*Lactococcus lactis* NZ9000 [32] and MG1363 [33] were grown at 30°C in M17 medium (BD Biosciences) supplemented with 0.5% glucose (GM17). *S. epidermidis* strains 9491 [18], 9 [20], HB [34] and K28 [18] were grown at 37°C in Tryptic Soy Broth (TSB) (BD Biosciences) supplemented with 0.25% glucose (TSBG). Strain 9491 is the prototype ATCC strain for SdrF; strain 9 is a clinical isolate from the forearm and has been shown to be defective in SdrF production [23],[35]; strain HB was obtained from a human patient with osteomyelitis; strain K28 is the prototype strain for SdrG [18]. *Escherichia coli* XL1-Blue (Stratagene) was used as an intermediate host in cloning experiments and was grown at 37°C in Luria Broth (LB) (BD Biosciences). Solid media was prepared by supplementing the corresponding liquid media with 1% agar. Strains harboring plasmids derived from pOri23 [31] were grown in the appropriate media supplemented with Erythromycin (Ery) (5 µg/mL for *L. lactis* and 500 µg/mL for *E. coli*) (Sigma). *L. lactis* constructs containing recombinant plasmids expressing full length SdrF and truncated forms of this protein have been previously described [23]. Antibodies directed against *S. epidermidis* proteins GehD and Fbe (homologous to SdrG) were kindly provided by Gabriela Bowden and Jan-Ingmar Flock respectively. Rabbit IgGs directed against the A and B domains of SdrF (anti-rASdrF and anti-rBSdrF, respectively) and control, pre-immune rabbit IgGs have been previously described [23].

### Construction of recombinant strains

Recombinant plasmids used in this study are described in Table 1. *L. lactis* strains containing recombinant plasmids harboring full length as well truncated forms of *sdrF* have been previously described [23]. Genes encoding *S. epidermidis* surface proteins were cloned into *L. lactis* NZ9000 using pORi23 as vector. *S. epidermidis* DNA fragments were obtained by PCR amplification with Platinum PCR Supermix High Fidelity (Invitrogen) according to the manufacturer's instructions. PCR products were purified with PCR Purification Kit (Qiagen), digested with the appropriate restriction endonucleases (Table 1) (New England Biolabs) and ligated to similarly digested pOri23. All cloning reactions were performed following manufacturer's instructions. The ligation mixture was used to transform *E. coli* XL1-Blue competent cells (Stratagene). Recombinant plasmids were assessed by restriction enzyme analysis and sequencing. Plasmids harboring the correct insert were purified and used to transform *L. lactis* NZ9000 and MG1363 competent cells as previously described [23],[33].

### Assessment of recombinant protein production

Recombinant protein production in *L. lactis* NZ9000 strains was assessed as follows; production of surface-exposed SdrF, SdrG and GehD was demonstrated as previously described by flow cytometry using IgG fractions purified from sera using the ImmunoPure IgG purification kit (Pierce) [23]. PIA production in *L. lactis* NZ9000 harboring pOri-icaO-47 was assessed as previously described with minor modifications [36]. Briefly, bacteria were cultured in microtiter plates (Nunc) for 24 hours. Wells were washed with phosphate-buffered saline (PBS) (Bio-Rad), air-dried and PIA was stained with 0.1% safranin.

### Driveline explantation and processing

For the VAD driveline explantation procedure, the driveline was cut from the device at surgery and was removed by pulling, avoiding manipulation of the internal part. The driveline was immediately rinsed in sterile, cold PBS, fixed in 10% formaldehyde for 30 min at room temperature and washed in PBS. The driveline was then cut longitudinally to create a flat surface and 6 mm-diameter disks were obtained with the use of a sterile cork borer for their subsequent use in adhesion assays.

### Non-implanted drivelines

Non-implanted DLs were provided by the manufacturer (Thoratec, Inc.) and were processed in the same manner as explanted DLs before their utilization in adherence assays.

### Bacterial adherence to drivelines

*L. lactis* or *S. epidermidis* cultures were grown to mid-log phase, harvested and resuspended in cold PBS, adjusted to OD_600_ = 0.1 corresponding to a count of 2–7×10^7^ CFU/mL and incubated for 1 hour at 37°C with disks obtained from explanted or non-implanted DLs. After incubation, disks were transferred to 50-ml Falcon tubes and extensively washed with PBS. Viable adherent bacteria were then lifted off the membrane using three 7-minute-incubations at 37°C with a solution of Trypsin-EDTA (1×) (Gibco). Bacterial suspensions were then plated onto appropriate culture media and incubated for 24–48 hours. To test the effect of antibodies on bacterial adherence to drivelines, *L. lactis* or *S. epidermidis* cell suspensions mentioned above were incubated for 45 min with appropriate concentrations of antibodies before adding the DL disks.

### Bacterial adherence to polystyrene surfaces

Binding of *L. lactis* and *S. epidermidis* to a polystyrene surface was measured as previously described with minor modifications [22]. Briefly, bacterial suspensions in PBS were adjusted to OD_600_ = 0.1 and incubated for 30 min at 37°C in polystyrene tissue culture Petri dishes (BD Biosciences) and washed five times with PBS. Adherent bacteria were stained with crystal violet and assessed by microscopic counting using a Nikon Eclipse E400 microscope. A 200×magnification was used. For each test, five random fields were counted using ImageJ 1.40 g software [37]. To measure antibody effect on adherence, bacteria were pre-incubated for 30 min with the appropriate concentration of purified IgGs before incubation in polystyrene dishes.

### DL histological analysis

Explanted DL samples were embedded in paraffin and sections (6 µm) were stained using Trichrome Stain (Masson) kit (Sigma) or Gram Staining kit (Sigma) in accordance with the manufacturer's instructions. Micrographs were taken using a Nikon Eclipse E400 microscope.

### Murine model of DL infection

C57BL/6J mice were divided into groups of 15 and transcutaneous drivelines were implanted following our recently described model [27]. Briefly, murine drivelines were constructed by coating silicone solid tubing with Dacron cloth. Mice were shaved and a 15 mm piece of murine driveline was transcutaneously implanted at the base of the neck. The internal (subcutaneous) part of the driveline spanned 10 mm; while the external part measured 5 mm. Mice were provided with food and water *ad libitum*. At the time of infection, drivelines were twisted to open the healing wound and the skin surrounding the DL inoculated with logarithmic phase bacterial cell suspensions in PBS (50 µl). When necessary, bacterial cell suspensions were pre-incubated for 30 min with the appropriate amount of antibodies prior to DL inoculation. Each mouse received 0.5–1×10^8^ CFU. The skin was covered with Tegaderm (3 M). Forty-eight hours after challenge the mice were euthanized, drivelines were explanted and their internal parts cut approximately 1 mm below the transcutaneous point. Samples of surrounding tissue were also obtained. Internal drivelines were transferred to a tube containing 1 mL sterile PBS and vortexed for 5 min. In order to ensure maximum release of adherent bacteria, the driveline was subjected to another vortexing cycle using fresh sterile PBS followed by incubation in a solution of Trypsin-EDTA (1×) (Gibco) for 7 min at 37°C. For each driveline all three samples were independently analyzed by serial dilution, plating onto the appropriate solid media, incubation and colony counting. The tissue was weighed, homogenized and plated onto agar. The results were expressed as the number of bacteria per gram of tissue.

### Statistical analysis

Statistical significance was determined using Student's t-test or analysis of variance. *P*<0.05 was considered to be statistically significant.
